# Supplementary material for: Multi-objective optimization of dual resource integrated scheduling problem of production equipment and RGVs considering conflict-free routing
Source: PLoS One. 2024 Jan 26;19(1):e0297139. doi: 10.1371/journal.pone.0297139 (PMC10834064; doi:10.1371/journal.pone.0297139)
Supplement: Attachment — Submitted filename: Response to Reviewers.docx [file pone.0297139.s006.docx]

**Response to Reviewers**

Journal: PLOS ONE

Manuscript ID: PONE-D-23-32956

Title: Multi-objective optimization of dual resource integrated scheduling problem of production equipment and RGVs considering conflict-free routing

Author(s): Qinglei Zhang, Jing Hu, Zhen Liu, Jianguo Duan

**Dear Ph.D Mazyar,**

We sincerely thank you and all reviewers for the valuable feedback that we have used to improve the quality of our manuscript.

The reviewer comments are laid out below in italicized, bold, and blue font. And specific concerns have been numbered. Given the questions and deficiencies raised, we have made major revisions. Our response is given in normal font and changes/additions to the manuscript are given in blue text. In addition, we have made corrections of English spelling and grammar, and we have revised the citation format as required.

In addition, since the full set of data used is listed in the article, we do not give additional data.

If there are any other modifications we could make, we would like very much to modify them. Thank you again for your positive comments on our manuscript.

We look forward to your reply as soon as possible.

With best wishes,

Yours sincerely,

Jing Hu

Corresponding author: Jing Hu

Email: janette10@163.com

**Reviewer #1:**

We sincerely thank you for your constructive questions and valuable comments. The changes/additions to the manuscript are given in blue text. Given the questions raised, our response is as follows.

1. ***“Abstract must be more precise with focus to objective, why the study is needed?”***

**Our response:**

The Abstract was carefully revised in the manuscript. At the beginning of the abstract, the current background and difficulties are described.

The revised abstract is as follows:

“*In flexible job shop scheduling problem (FJSP), the collision of bidirectional rail guided vehicles (RGVs) directly affects RGVs scheduling, and it is closely coupled with the allocation of production equipment, which directly affects the production efficiency. In this problem, taking minimizing the maximum completion time of RGVs and minimizing the maximum completion time of products as multi-objectives a dual-resource integrated scheduling model of production equipment and RGVs considering conflict-free routing problem (CFRP) is proposed. To solve the model, a multi-objective improved discrete grey wolf optimizer (MOID-GWO) is designed. Further, the performance of popular multi-objective evolutionary algorithms (MOEAs) such as NSGA-Ⅱ, SPEA2 and MOPSO are selected for comparative test. The results show that, among 42 instances of different scales designed, 37, 34 and 28 instances in MOID-GWO are superior to the comparison algorithms in metrics of generational distance (GD), inverted GD (IGD) and Spread, respectively. Moreover, in metric of Convergence and Diversity (CD), the Pareto frontier (PF) obtained by MOID-GWO is closer to the optimal solution. Finally, taking the production process of a construction machinery equipment component as an example, the validity and feasibility of the model and algorithm are verified.*”

2. ***“Brief results must be shown in Abstract and at present it is missing.”***

**Our response:**

We present our results in a revised Abstract. We have given the summary in its entirety in *question* 1, and the results shown in Abstract are shown here.

“*The results show that, among 42 instances of different scales designed, 37, 34 and 28 instances in MOID-GWO are superior to the comparison algorithms in metrics of generational distance (GD), inverted GD (IGD) and Spread, respectively. Moreover, in metric of Convergence and Diversity (CD), the Pareto frontier (PF) obtained by MOID-GWO is closer to the optimal solution. Finally, taking the production process of a construction machinery equipment component as an example, the validity and feasibility of the model and algorithm are verified.*”

3. ***“Why you have used Grey Wolf algorithm? Why not other heuristic algorithms like SA, hill-climbing algorithm, GA, ABC, etc.? Discuss advantages of these algorithms.”***

**Our response:**

In Introduction and the evolutionary algorithm section of the Literature Review, we expounded the advantages of the Grey Wolf Optimizer (GWO).

“*Grey wolf optimizer (GWO) has good performance in both convergence speed and solving accuracy, but the original algorithm is mainly used to solve continuous and single-objective optimization problems^[6]^*” can be found in Introduction.

Further, to illustrate the advantages of GWO, we added a table in Literature Review. This table aims to compare the global search ability, convergence speed, simplicity and ease of implementation, and applicability of complex constraints. The revision is as follows:

“*The current widely used evolutionary algorithms (EA)^[24]^ are shown in Tab. 1, which comprehensively considers the global search ability, Rate of convergence, simplicity and ease of implementation, applicability of complex constraint. Although PSO, EA, GA and ABC perform well in Simplicity and ease of implementation and complex constraint applicability, many parameter adjustments are involved, resulting in unstable results.*”

Tab. 1 Comparison of popular evolutionary algorithms

| Evolutionary algorithms | Global search capability | Rate of convergence | Simplicity and ease of implementation | Applicability of complex constraints |
| --- | --- | --- | --- | --- |
| GWO | Strong | Fast | Facile | Applicative |
| Simulated Annealing (SA)^[27]^ | Moderate | Slow | Facile | Hard |
| Hill-climbing | Weak | Slow | Facile | Hard |
| GA | Moderate | Moderate | Moderate | Moderate |
| Artificial Bee Colony (ABC)^[28]^ | Strong | Moderate | Moderate | Moderate |
| PSO | Moderate | Moderate | Moderate | Moderate |
| Differential Evolution (DE) | Moderate | Moderate | Moderate | Moderate |

4. ***“You should extend the literature review with application of heuristic algorithms and discuss them to show gap. Remove papers published before 2018. I suggest authors to read and discuss below interesting papers:***

***Das, M., Roy, A., Maity, S., Kar, S., & Sengupta, S. . (2022). Solving fuzzy dynamic ship routing and scheduling problem through new genetic algorithm. Decision Making: Applications in Management and Engineering, 5(2), 329–361.;***

***Mzili, I., Mzili, T., & Riffi , M. E. (2023). Efficient routing optimization with discrete penguins search algorithm for MTSP. Decision Making: Applications in Management and Engineering, 6(1), 730–743.;***

***Chaki, S., & Bose, D. . (2022). Optimization of spot-welding process using Taguchi based Cuckoo search algorithm. Decision Making: Applications in Management and Engineering, 5(2), 316–328.”***

**Our response:**

We deleted most of the literature published before 2018 and added the literature of recent years. Meanwhile, we carefully read the three provided interesting articles. The first two papers are the optimization of TSP problems, and the third is about continuous optimization problems. We add the algorithms of these three papers to the literature review.

5. ***“Provide better validation section with comparisons with the existing models. Add more discussion on the results and discuss advantages and limitations.”***

**Our response:**

We added a new chapter to the original 5.1 and 5.2 sections. This chapter effectively compares the improved strategy adopted and the original algorithm without the improved strategy. The results show that the algorithm can effectively improve the quality and diversity of the initial solution, and then improve the quality of the final Pareto solution. The newly added chapters are shown below:

## “*5.2 Effectiveness analysis of the improvement strategy*

*To evaluate the effectiveness of the proposed RPTL strategy, we conducted a comparison experiment between MOID-GWO and MOID-GWO_r_ (MOID-GWO using a random initial population strategy). In MOID-GWO_r_, other parameters are consistent except the initial population strategy.*

*Since spread(SP), generational distance(GD) and inverted GD(IGD) can effectively evaluate the diversity and overall performance of multi-objective evolutionary algorithms (MOEAs), these three metrics are used to evaluate the effectiveness of RPTL strategies.*

*Two algorithms are independently run 30 times on 42 instances of different scales, and the results of average value are shown in Tab. 4. The best values of each metric in different algorithms are shown in bold.*

*It can be seen from the results that MOID-GWO obtained the best value of SP in 29 instances, indicating that the strategy can effectively maintain the diversity of the population. MOID-GWO obtains the best value of GD in 32 instances, which means that MOID-GWO has better convergence than MOID-GWO_r_ in most instances. Most notably, MOID-GWO obtains the best value of IGD in 36 instances, which shows that MOID-GWO is superior to MOID-GWOr in diversity and convergence.*

*In sum, RPTL initializing population strategy is helpful to improve the accuracy of the algorithm Effectiveness analysis showed that RPTL can help the MOID-GWO improve the quality of the initial solution, and achieve better accuracy ultimately.*

Tab. 4 Effectiveness analysis of RPTL

| Problem | SP | | GD | | IGD | |
| --- | --- | --- | --- | --- | --- | --- |
|  | MOID-GWO_r_ | MOID-GWO | MOID-GWO_r_ | MOID-GWO | MOID-GWO_r_ | MOID-GWO |
| n5m5 | 1.18E-01 | **1.05E-01** | 3.14E-02 | **1.08E-02** | 4.52E-02 | **4.23E-02** |
| n10m5 | 1.23E+00 | **8.96E-01** | 7.41E-02 | **7.02E-02** | 1.43E-02 | **1.02E-02** |
| n20m5 | **6.02E-01** | 6.72E-01 | 6.87E-02 | **6.49E-02** | **1.33E-02** | 4.78E-02 |
| n40m5 | 1.37E-01 | **1.22E-01** | **2.29E-02** | 2.45E-02 | 4.81E-02 | **1.95E-02** |
| n60m5 | **8.73E-01** | 9.39E-01 | **7.80E-02** | 8.90E-02 | 4.94E-02 | **3.49E-02** |
| n80m5 | **9.61E-01** | 1.16E+00 | 8.93E-02 | **6.29E-02** | 1.45E-02 | **2.46E-02** |
| n5m6 | 8.91E-01 | **8.56E-01** | 3.22E-02 | **1.15E-02** | 7.89E-02 | **3.62E-02** |
| n10m6 | **6.65E-01** | 7.32E-01 | 2.84E-02 | **2.23E-02** | 1.46E-01 | **2.63E-02** |
| n20m6 | 1.39E+00 | **8.29E-01** | **2.25E-02** | 4.36E-02 | **6.00E-02** | 7.36E-02 |
| n40m6 | 7.55E-01 | **6.87E-01** | 5.76E-02 | **5.08E-02** | 5.67E-02 | **4.83E-02** |
| n60m6 | 8.87E-01 | **8.59E-01** | 3.90E-02 | **2.31E-02** | 8.15E-02 | **7.68E-02** |
| n80m6 | **6.01E-01** | 7.05E-01 | **4.35E-02** | 5.11E-02 | 1.75E-02 | **1.21E-02** |
| n5m7 | 8.92E-01 | **8.21E-01** | 6.30E-02 | **4.55E-02** | 5.40E-02 | **3.57E-02** |
| n10m7 | 3.34E-01 | **2.65E-01** | **3.86E-02** | 4.05E-02 | **7.81E-03** | 8.86E-03 |
| n20m7 | **6.02E-01** | 6.12E-01 | 3.47E-02 | **2.58E-02** | 4.43E-02 | **3.81E-02** |
| n40m7 | 6.71E-01 | **5.79E-01** | **8.29E-02** | 8.72E-02 | 9.53E-02 | **2.85E-03** |
| n60m7 | 9.20E-01 | **7.42E-01** | **5.28E-02** | 6.12E-02 | 7.24E-02 | **7.08E-02** |
| n80m7 | 4.84E-01 | **4.52E-01** | 2.95E-02 | **2.83E-02** | 9.64E-02 | **2.49E-03** |
| n5m8 | **8.44E-01** | 8.16E-01 | **2.66E-02** | 2.16E-03 | 8.92E-02 | **8.44E-02** |
| n10m8 | 0.84E-01 | **7.90E-01** | 5.89E-02 | **5.16E-02** | 8.47E-02 | **5.60E-02** |
| n20m8 | 6.45E-01 | **5.78E-01** | 4.99E-02 | **2.52E-02** | 6.81E-02 | **6.21E-01** |
| n40m8 | **6.12E-01** | 6.43E-01 | 2.48E-02 | **2.40E-02** | **5.02E-03** | 5.59E-02 |
| n60m8 | 8.83E-01 | **7.80E-01** | 5.99E-02 | **5.46E-02** | 7.59E-02 | **8.60E-02** |
| n80m8 | 7.85E-01 | **6.21E-01** | 7.54E-02 | **7.05E-02** | 9.04E-02 | **8.11E-02** |
| n5m9 | **8.05E-01** | 8.20E-01 | **6.38E-02** | 9.97E-02 | 1.40E-02 | **2.55E-02** |
| n10m9 | 8.01E-01 | **7.18E-01** | **3.49E-02** | 4.49E-02 | 1.04E-02 | **1.11E-02** |
| n20m9 | 8.06E-01 | **7.30E-01** | 8.26E-02 | **7.72E-02** | 5.32E-03 | **4.15E-03** |
| n40m9 | 1.14E+00 | **8.28E-01** | **5.75E-02** | 6.72E-02 | 6.36E-02 | **8.07E-02** |
| n60m9 | **7.02E-01** | 9.21E-01 | **1.04E-02** | 2.51E-02 | **2.00E-02** | 3.21E-02 |
| n80m9 | 5.57E-01 | **5.09E-01** | 9.61E-03 | **8.84E-03** | 2.01E-02 | **1.50E-02** |
| n5m10 | 5.85E-01 | **4.55E-01** | 8.92E-03 | **7.74E-03** | 2.41E-02 | **1.07E-02** |
| n10m10 | **8.28E-01** | 9.28E-01 | 6.72E-02 | **5.63E-02** | 4.63E-02 | **3.73E-02** |
| n20m10 | **4.51E-01** | 9.10E-01 | 5.94E-02 | **4.53E-02** | **6.47E-02** | 8.76E-02 |
| n40m10 | 7.58E-01 | **5.48E-01** | 6.19E-02 | **5.09E-02** | 9.42E-02 | **7.12E-02** |
| n60m10 | 9.24E-01 | **7.17E-01** | **4.28E-02** | 5.71E-02 | 3.99E-02 | **3.78E-03** |
| n80m10 | 9.10E-01 | **7.75E-01** | 4.22E-02 | **2.78E-02** | 6.72E-02 | **5.41E-02** |
| n5m11 | 1.04E+00 | **8.38E-01** | 9.16E-02 | **8.38E-02** | 4.37E-02 | **4.23E-02** |
| n10m11 | 8.31E-01 | **7.60E-01** | 3.88E-02 | **3.53E-02** | 1.49E-02 | **1.46E-02** |
| n20m11 | 8.92E-01 | **7.08E-01** | 6.19E-02 | **5.14E-02** | 1.02E-02 | **7.59E-03** |
| n40m11 | **8.33E-01** | 9.55E-01 | 3.45E-02 | **3.23E-02** | 3.10E-03 | **2.50E-03** |
| n60m11 | 8.45E-01 | **6.92E-01** | 7.31E-01 | **6.98E-02** | 1.72E-02 | **1.33E-02** |
| n80m11 | 9.22E-01 | **7.21E-01** | 2.15E-02 | **1.50E-02** | 5.22E-02 | **3.38E-02** |
| Hit rate | 13/42 | 29/42 | 10/42 | 32/42 | 6/42 | 36/42 |

6. ***“Comparison can be made effective too with more results and discussion and also expand the experimental setup.”***

**Our response:**

We have added some new contents and experiments to further verify the effectiveness of the proposed algorithm. The specific changes are given in question 5.

7. ***“- Limitations - Addressing your research limitations could enhance the credibility, applicability, and impact of your research. It is important to note that limitations in a research paper do not necessarily imply negative aspects but rather areas that offer opportunities for further refinement and improvement. Identifying and discussing these limitations transparently can contribute to the overall growth and effectiveness of the study. Explicitly acknowledge the limitations of the proposed framework and model. Address any potential drawbacks or constraints and how they were managed or could be improved in future iterations.”***

**Our response:**

In the last chapter, we present the limitations of the model and algorithm in this paper. The specific changes are as follows:

“*Of course, the proposed models and algorithms are limited to some extent. In terms of the model, it is assumed that once the production equipment starts working, the work will not stop. It has weak robustness and anti-interference to the disturbance caused by sudden events. Therefore, dynamic perturbations, such as new workpiece joining, machine failure, will be considered in DRISP-PERCFR in the future. In terms of the algorithm, although the three-chain encoding of MOID-GWO improves the quality of solution, it increases the complexity for the subsequent, so new codec forms will be studied in the future.*”

8. ***“End the paper with a section on potential future research directions. Highlight areas where further studies could extend or build upon your work.”***

**Our response:**

We have carefully revised the last chapter. At the same time of revising the conclusions of this paper, the future research direction is pointed out. The specific changes are as follows.

“*In the manufacturing process, CFRP between RGVs increases the complexity of RGVs and production equipment integration scheduling. Therefore, the dual resource integration scheduling of production equipment and RGVs considering CFRP is studied,, and the following conclusions are obtained:*

*1) DRISP-PERCFR is established. The conflict is divided into loading and unloading conflict, waiting occupation conflict and transportation conflict, and the solution of the conflict with site occupation update is proposed respectively, which improves the accurate mapping degree of the model to the reality.*

*(2) MOID-GWO is proposed for the characteristics of DRISP-PERCFR. MOID-GWO improves the quality and diversity of the initial solution, and solves the dependence degree of original GWO on the initial solution.*

*(3) The feasibility and effectiveness of the proposed model and algorithm are verified in different scale tests and the actual production process of the engineering machinery equipment manufacturing workshop.*

*Of course, the proposed models and algorithms are limited to some extent. In terms of the model, it is assumed that once the production equipment starts working, the work will not stop. It has weak robustness and anti-interference to the disturbance caused by sudden events. Therefore, dynamic perturbations, such as new workpiece joining, machine failure, will be considered in DRISP-PERCFR in the future. In terms of the algorithm, although the three-chain encoding of MOID-GWO improves the quality of solution, it increases the complexity for the subsequent, so new codec forms will be studied in the future.*”

**Reviewer #2:**

We sincerely thank you for your constructive questions and valuable comments. Given the questions raised, our response is as follows.

1. ***“The abstract lacks focus and clarity; it also doesn’t include the motivation for the proposed methods.”***

**Our response:**

We have revised the Abstract and explained the motivation of the method. Moreover, we have included important results. The revised Abstract is as follows:

“*In flexible job shop scheduling problem (FJSP), the collision of bidirectional rail guided vehicles (RGVs) directly affects RGVs scheduling, and it is closely coupled with the allocation of production equipment, which directly affects the production efficiency. In this problem, taking minimizing the maximum completion time of RGVs and minimizing the maximum completion time of products as multi-objectives a dual-resource integrated scheduling model of production equipment and RGVs considering conflict-free routing problem (CFRP) is proposed. To solve the model, a multi-objective improved discrete grey wolf optimizer(MOID-GWO) is designed. Further, the performance of popular multi-objective evolutionary algorithms (MOEAs) such as NSGA-Ⅱ, SPEA2 and MOPSO are selected for comparative test. The results show that, among 42 instances of different scales designed, 37, 34 and 28 instances in MOID-GWO are superior to the comparison algorithms in metrics of generational distance (GD), inverted GD (IGD) and Spread, respectively. Moreover, in metric of Convergence and Diversity (CD), the Pareto frontier (PF) obtained by MOID-GWO is closer to the optimal solution. Finally, taking the production process of a construction machinery equipment component as an example, the validity and feasibility of the model and algorithm are verified.*”

2. ***“The structure of the introduction is not in a standard form. first, we need to find the basics of your problems and then the main aspects and issues related to them. Do not pay attention to lots of details (especially the foundation of the problem)”***

**Our response:**

We have revised the introduction. In the introduction, we point out the background of the problem, the current research gaps and the significance of studying the problem.

The revised Introduction is as follows:

“*With the promotion of Industry 4.0, to improve production efficiency under the demand of batch and individualized production, two or more single-load bi-directional rail guided vehicles (RGVs) are often used to transport large-size and large-tonnage products and components on the single track^[1]^.*

*At present, the existence of rail occupying and bi-directional conflict between RGVS leads to the occurrence of delayed loading and unloading problems. Although the flexible job-shop scheduling problem(FJSP)^[2]^ research is extensive, most of them do not consider the transportation time, or treat the transportation time between workstations as a constant value, which is a weak guide for the actual production units where RGVs undertake logistics and transport.*

*Further, the scheduling of RGVs directly affects the change of production equipment scheduling. Although some scholars have focused on RGV scheduling optimization，such as considering the conditions of energy constraint^[3]^, speed constraint^[4]^ and conflict constraint^[5]^. However, adding RGV conflict constraints to FJSP will significantly increase the complexity of the problem. Therefore, there is little research on dual resource and multi-objective integrated scheduling of RGV and production equipment. To solve the problem of delayed loading and unloading in the manufacturing process caused by route conflict between RGVs, and improve the production efficiency of integrated operation between RGV and production equipment, the multi-objective optimization of dual resource integrated scheduling problem of production equipment and RGVs considering conflict-free routing (DRISP-PERCFR) is researched, and a non-linear integer programming model is developed. Grey wolf optimizer (GWO) has good performance in both convergence speed and solving accuracy, but the original algorithm is mainly used to solve continuous and single-objective optimization problems^[6]^. A new multi-objective improved discrete GWO (MOID-GWO) is designed to solve the model and tested in different instances. The main innovations are as follows:*

*(1) The integrated scheduling model of DRISP-PERCFR is established, and a new RGVs conflict avoidance solution is proposed in the coupling relationship between transportation and production The route conflicts of RGVs are described more detail, and the loading/unloading occupancy conflict, waiting occupancy conflict and transportation conflict models are constructed and solved respectively.*

*(2) MOID-GWO is proposed to solve the above problems. To improve the quality and diversity of the initial population, The reducing job process time, transportation time, loading and unloading time strategy (RPTL), and the improved auction protocol heuristic strategy (IAPH) are developed to solve the dependence of GWO on the quality and diversity of initial solutions.*

*(3) In MOID-GWO, an improved discrete combinational optimization search operator is designed to update the individual location of grey wolves, and the local search capability is strengthened by designing a neighbour structure.*

*(4) By constructing different scale test examples, the performance index of Pareto optimal solution is compared with NSGA-Ⅱ, strength Pareto evolutionary algorithm 2 (SPEA2), and multi-objective particle swarm optimization algorithm (MOPSO).*

*(5) The effectiveness of DRISP-PERCFR’s model and MOID-GWO is verified by a practical case of a welding manufacturing enterprise.*

*The rest of the paper is organized as follows: Section 2 provides a literature review related to the problem. In Section 3, related problems are explained and mathematical models are established. In Section 4, MOID-GWO is designed. In Section 5, experimental research is carried out. Anda practical case study is analyzed in Section 6. Conclusions and future work are introduced in Section 7.*”

3. ***“Improve the literature review. Add several pieces of research in 2019-2023. Moreover, the following references should be used:***

***A robust possibilistic programming framework for designing an organ transplant supply chain under uncertainty. Annals of Operations Research, 1-38.***

***Two-echelon electric vehicle routing problem with a developed moth-flame meta-heuristic algorithm. Operations Management Research, 1-22.***

***Efficient multi-objective meta-heuristic algorithms for energy-aware non-permutation flow-shop scheduling problem. Expert Systems with Applications, 213, 119077.***

***Designing a portfolio-based closed-loop supply chain network for dairy products with a financial approach: Accelerated Benders decomposition algorithm. Computers & Operations Research, 155, 106244.***

***Integration of blockchain-enabled closed-loop supply chain and robust product portfolio design. Computers & Industrial Engineering, 179, 109211.***

***Just-in-time scheduling in identical parallel machine sequence-dependent group scheduling problem. Journal of Industrial & Management Optimization.”***

**Our response:**

We have carefully revised the literature review section, removing most of the pre-2018 articles and adding more recent literatures.

Moreover, we carefully reviewed the interesting papers offered. References 1, 2, and 5 are based on supply chain issues or location issues. This paper is mainly about the conflict free routing problem between job scheduling and RGV in the manufacturing shop, so these three papers are not suitable for reference in this paper. But 3,4 and 6 are very instructive for us in job shop scheduling problems and algorithms. Thus, 3, 4, and 6 are cited, which can be found in references [16],[30],[33] in revised paper.

4. ***“Most of the methodological choices lack a clear motivation, and their impact on performance is not analyzed on the manuscript. On the whole, there is no clear indication of where the authors see the main innovation and value of the methodology described.”***

**Our response:**

We appreciate your valuable comments. There are indeed lapses in our expression in the process of method selection. Therefore, we add some explanations before presenting the model and method in manuscript.

For example, we added an explanation of the reason and significance of studying conflict free routing problems in integrated scheduling in Introduction. The explanation is as follows:

“*At present, the existence of rail occupying and bi-directional conflict between RGVS leads to the occurrence of delayed loading and unloading problems. Although the flexible job-shop scheduling problem(FJSP)^[2]^ research is extensive, most of them do not consider the transportation time, or treat the transportation time between workstations as a constant value, which is a weak guide for the actual production units where RGVs undertake logistics and transport.*”

In this paper, GWO is selected as the basic algorithm, and it is improved to solve the model. We added the advantages of GWO in Introduction, and added a new comparison table of evolutionary algorithms in Literature Review. In the table, we analyzed the global search ability, convergence performance, simplicity and ease of implementation and the application performance of complex constraints of multiple algorithms. Through comprehensive evaluation, GWO is selected as the basic algorithm. Specific, as shown below.

**In Introduction:**

“*Grey wolf optimizer (GWO) has good performance in both convergence speed and solving accuracy, but the original algorithm is mainly used to solve continuous and single-objective optimization problems^[6]^. A new multi-objective improved discrete GWO (MOID-GWO) is designed to solve the model and tested in different instances*.”

**In Literature Review:**

“*The current widely used evolutionary algorithms (EA)^[24]^ are shown in Tab. 1, which comprehensively considers the global search ability, Rate of convergence, simplicity and ease of implementation, applicability of complex constraint. Although PSO, EA, GA and ABC perform well in Simplicity and ease of implementation and complex constraint applicability, many parameter adjustments are involved, resulting in unstable results.*

*Compared with other algorithms, grey wolf optimizer (GWO) has better performance in terms of solution quality and stability, and has been widely used^[25]^. The original GWO flow can be found in [26].* ”

Tab. 1 Comparison of popular evolutionary algorithms

| Evolutionary algorithms | Global search capability | Rate of convergence | Simplicity and ease of implementation | Applicability of complex constraints |
| --- | --- | --- | --- | --- |
| GWO | Strong | Fast | Facile | Applicative |
| Simulated Annealing (SA)^[27]^ | Moderate | Slow | Facile | Hard |
| Hill-climbing | Weak | Slow | Facile | Hard |
| GA | Moderate | Moderate | Moderate | Moderate |
| Artificial Bee Colony (ABC)^[28]^ | Strong | Moderate | Moderate | Moderate |
| PSO | Moderate | Moderate | Moderate | Moderate |
| Differential Evolution (DE) | Moderate | Moderate | Moderate | Moderate |

Since GWO depends on the initial value to some extent, we improve the population initialization method. In the chapter on population initialization, we indicate the purpose of method.

“*To ensure the quality and diversity of the initial population, random initialization, RPTL, and IAPH are used to initialize the population.*” The RPTL method is proposed according to the model and codec features.

5. ***“There is no conceptual comparison with existing approaches, no discussion on the benefits and drawbacks of the new approach. Thus discussions and comparative analyses should be added, also it is important to compare your method with literature ones.”***

**Our response:**

We added a new chapter to the original 5.1 and 5.2 sections. This chapter effectively compares the improved strategy adopted and the original algorithm without the improved strategy. The results show that the algorithm can effectively improve the quality and diversity of the initial solution, and then improve the quality of the final Pareto solution. The newly added chapters are shown below:

## “*5.2 Effectiveness analysis of the improvement strategy*

*To evaluate the effectiveness of the proposed RPTL strategy, we conducted a comparison experiment between MOID-GWO and MOID-GWO_r_ (MOID-GWO using a random initial population strategy). In MOID-GWO_r_, other parameters are consistent except the initial population strategy.*

*Since spread(SP), generational distance(GD) and inverted GD(IGD) can effectively evaluate the diversity and overall performance of multi-objective evolutionary algorithms (MOEAs), these three metrics are used to evaluate the effectiveness of RPTL strategies.*

*Two algorithms are independently run 30 times on 42 instances of different scales, and the results of average value are shown in Tab. 4. The best values of each metric in different algorithms are shown in bold.*

*It can be seen from the results that MOID-GWO obtained the best value of SP in 29 instances, indicating that the strategy can effectively maintain the diversity of the population. MOID-GWO obtains the best value of GD in 32 instances, which means that MOID-GWO has better convergence than MOID-GWO_r_ in most instances. Most notably, MOID-GWO obtains the best value of IGD in 36 instances, which shows that MOID-GWO is superior to MOID-GWOr in diversity and convergence.*

*In sum, RPTL initializing population strategy is helpful to improve the accuracy of the algorithm Effectiveness analysis showed that RPTL can help the MOID-GWO improve the quality of the initial solution, and achieve better accuracy ultimately.*

Tab. 4 Effectiveness analysis of RPTL

| Problem | SP | | GD | | IGD | |
| --- | --- | --- | --- | --- | --- | --- |
|  | MOID-GWO_r_ | MOID-GWO | MOID-GWO_r_ | MOID-GWO | MOID-GWO_r_ | MOID-GWO |
| n5m5 | 1.18E-01 | **1.05E-01** | 3.14E-02 | **1.08E-02** | 4.52E-02 | **4.23E-02** |
| n10m5 | 1.23E+00 | **8.96E-01** | 7.41E-02 | **7.02E-02** | 1.43E-02 | **1.02E-02** |
| n20m5 | **6.02E-01** | 6.72E-01 | 6.87E-02 | **6.49E-02** | **1.33E-02** | 4.78E-02 |
| n40m5 | 1.37E-01 | **1.22E-01** | **2.29E-02** | 2.45E-02 | 4.81E-02 | **1.95E-02** |
| n60m5 | **8.73E-01** | 9.39E-01 | **7.80E-02** | 8.90E-02 | 4.94E-02 | **3.49E-02** |
| n80m5 | **9.61E-01** | 1.16E+00 | 8.93E-02 | **6.29E-02** | 1.45E-02 | **2.46E-02** |
| n5m6 | 8.91E-01 | **8.56E-01** | 3.22E-02 | **1.15E-02** | 7.89E-02 | **3.62E-02** |
| n10m6 | **6.65E-01** | 7.32E-01 | 2.84E-02 | **2.23E-02** | 1.46E-01 | **2.63E-02** |
| n20m6 | 1.39E+00 | **8.29E-01** | **2.25E-02** | 4.36E-02 | **6.00E-02** | 7.36E-02 |
| n40m6 | 7.55E-01 | **6.87E-01** | 5.76E-02 | **5.08E-02** | 5.67E-02 | **4.83E-02** |
| n60m6 | 8.87E-01 | **8.59E-01** | 3.90E-02 | **2.31E-02** | 8.15E-02 | **7.68E-02** |
| n80m6 | **6.01E-01** | 7.05E-01 | **4.35E-02** | 5.11E-02 | 1.75E-02 | **1.21E-02** |
| n5m7 | 8.92E-01 | **8.21E-01** | 6.30E-02 | **4.55E-02** | 5.40E-02 | **3.57E-02** |
| n10m7 | 3.34E-01 | **2.65E-01** | **3.86E-02** | 4.05E-02 | **7.81E-03** | 8.86E-03 |
| n20m7 | **6.02E-01** | 6.12E-01 | 3.47E-02 | **2.58E-02** | 4.43E-02 | **3.81E-02** |
| n40m7 | 6.71E-01 | **5.79E-01** | **8.29E-02** | 8.72E-02 | 9.53E-02 | **2.85E-03** |
| n60m7 | 9.20E-01 | **7.42E-01** | **5.28E-02** | 6.12E-02 | 7.24E-02 | **7.08E-02** |
| n80m7 | 4.84E-01 | **4.52E-01** | 2.95E-02 | **2.83E-02** | 9.64E-02 | **2.49E-03** |
| n5m8 | **8.44E-01** | 8.16E-01 | **2.66E-02** | 2.16E-03 | 8.92E-02 | **8.44E-02** |
| n10m8 | 0.84E-01 | **7.90E-01** | 5.89E-02 | **5.16E-02** | 8.47E-02 | **5.60E-02** |
| n20m8 | 6.45E-01 | **5.78E-01** | 4.99E-02 | **2.52E-02** | 6.81E-02 | **6.21E-01** |
| n40m8 | **6.12E-01** | 6.43E-01 | 2.48E-02 | **2.40E-02** | **5.02E-03** | 5.59E-02 |
| n60m8 | 8.83E-01 | **7.80E-01** | 5.99E-02 | **5.46E-02** | 7.59E-02 | **8.60E-02** |
| n80m8 | 7.85E-01 | **6.21E-01** | 7.54E-02 | **7.05E-02** | 9.04E-02 | **8.11E-02** |
| n5m9 | **8.05E-01** | 8.20E-01 | **6.38E-02** | 9.97E-02 | 1.40E-02 | **2.55E-02** |
| n10m9 | 8.01E-01 | **7.18E-01** | **3.49E-02** | 4.49E-02 | 1.04E-02 | **1.11E-02** |
| n20m9 | 8.06E-01 | **7.30E-01** | 8.26E-02 | **7.72E-02** | 5.32E-03 | **4.15E-03** |
| n40m9 | 1.14E+00 | **8.28E-01** | **5.75E-02** | 6.72E-02 | 6.36E-02 | **8.07E-02** |
| n60m9 | **7.02E-01** | 9.21E-01 | **1.04E-02** | 2.51E-02 | **2.00E-02** | 3.21E-02 |
| n80m9 | 5.57E-01 | **5.09E-01** | 9.61E-03 | **8.84E-03** | 2.01E-02 | **1.50E-02** |
| n5m10 | 5.85E-01 | **4.55E-01** | 8.92E-03 | **7.74E-03** | 2.41E-02 | **1.07E-02** |
| n10m10 | **8.28E-01** | 9.28E-01 | 6.72E-02 | **5.63E-02** | 4.63E-02 | **3.73E-02** |
| n20m10 | **4.51E-01** | 9.10E-01 | 5.94E-02 | **4.53E-02** | **6.47E-02** | 8.76E-02 |
| n40m10 | 7.58E-01 | **5.48E-01** | 6.19E-02 | **5.09E-02** | 9.42E-02 | **7.12E-02** |
| n60m10 | 9.24E-01 | **7.17E-01** | **4.28E-02** | 5.71E-02 | 3.99E-02 | **3.78E-03** |
| n80m10 | 9.10E-01 | **7.75E-01** | 4.22E-02 | **2.78E-02** | 6.72E-02 | **5.41E-02** |
| n5m11 | 1.04E+00 | **8.38E-01** | 9.16E-02 | **8.38E-02** | 4.37E-02 | **4.23E-02** |
| n10m11 | 8.31E-01 | **7.60E-01** | 3.88E-02 | **3.53E-02** | 1.49E-02 | **1.46E-02** |
| n20m11 | 8.92E-01 | **7.08E-01** | 6.19E-02 | **5.14E-02** | 1.02E-02 | **7.59E-03** |
| n40m11 | **8.33E-01** | 9.55E-01 | 3.45E-02 | **3.23E-02** | 3.10E-03 | **2.50E-03** |
| n60m11 | 8.45E-01 | **6.92E-01** | 7.31E-01 | **6.98E-02** | 1.72E-02 | **1.33E-02** |
| n80m11 | 9.22E-01 | **7.21E-01** | 2.15E-02 | **1.50E-02** | 5.22E-02 | **3.38E-02** |
| Hit rate | 13/42 | 29/42 | 10/42 | 32/42 | 6/42 | 36/42 |

6. ***“Conclusion is so brief and it did not conduct all aspects of your research well. for example, limitations and future directions are not prepared well.”***

**Our response:**

We have carefully revised the last chapter. At the same time of revising the conclusions of this paper, the future research direction is pointed out. The specific changes are as follows.

“*In the manufacturing process, CFRP between RGVs increases the complexity of RGVs and production equipment integration scheduling. Therefore, the dual resource integration scheduling of production equipment and RGVs considering CFRP is studied,, and the following conclusions are obtained:*

*1) DRISP-PERCFR is established. The conflict is divided into loading and unloading conflict, waiting occupation conflict and transportation conflict, and the solution of the conflict with site occupation update is proposed respectively, which improves the accurate mapping degree of the model to the reality.*

*(2) MOID-GWO is proposed for the characteristics of DRISP-PERCFR. MOID-GWO improves the quality and diversity of the initial solution, and solves the dependence degree of original GWO on the initial solution.*

*(3) The feasibility and effectiveness of the proposed model and algorithm are verified in different scale tests and the actual production process of the engineering machinery equipment manufacturing workshop.*

*Of course, the proposed models and algorithms are limited to some extent. In terms of the model, it is assumed that once the production equipment starts working, the work will not stop. It has weak robustness and anti-interference to the disturbance caused by sudden events. Therefore, dynamic perturbations, such as new workpiece joining, machine failure, will be considered in DRISP-PERCFR in the future. In terms of the algorithm, although the three-chain encoding of MOID-GWO improves the quality of solution, it increases the complexity for the subsequent, so new codec forms will be studied in the future.*”
